# Obstructive sleep apnea (OSA) screening and risk of postoperative complications in adult surgical patients: differences between the STOP-Bang and Step-2 scoring strategy

**DOI:** 10.1186/s12871-026-03900-1

**Published:** 2026-05-13

**Authors:** Martin Roesslein, Paula Ortiz-Lucas, Hartmut Buerkle, Torsten Loop, Axel Semmelmann

**Affiliations:** 1 Department of Anesthesiology and Critical Care, Medical Center-University of Freiburg, Faculty of Medicine, University of Freiburg, Freiburg, 79106 Germany; 2 Surgery in Stuehlinger, Freiburg, 79106 Germany

**Keywords:** Obstructive sleep apnea (OSA), STOP-bang score, Postoperative complications

## Abstract

**Purpose:**

Obstructive sleep apnea (OSA) is associated with an increased risk of postoperative complications. However, data on the relationship between previously undiagnosed high-risk patients and postoperative outcomes remain limited. The study aimed to evaluate the association between OSA risk-assessed using the STOP-Bang score (SBS) and the Step-2 scoring strategy – and the incidence of postoperative complications as well as prolonged hospital length of stay.

**Methods:**

A total of 4,292 adult patients undergoing elective, non-cardiac surgery between 2015 to 2016 were included. All patients underwent preoperative assessment for OSA risk using the STOP-Bang score questionnaire and the Step-2 algorithm. Patients were stratified into low-risk (SBS 0—2) and high-risk (HR) groups, defined as either (SBS 5—8 (S1HR) or Step-2 high-risk (S2HR). The primary outcome was the occurrence of major postoperative complications. Secondary outcomes included prolonged hospital length of stay.

**Results:**

Patients classified as high-risk for OSA exhibited a significant increased risk of major postoperative complications compared with the low-risk group (odds ratio [OR]: 1.65 (95% CI 1.09—2.48); S2HR vs. low-risk: OR 2.17 (95% CI 1.57—3.01). No significant difference in complications risk was observed between the two high-risk stratification methods (OR 0.96 (95% CI 0.64—1.33; *P* = 0.8). Similarly, high-risk patients were more likely to experience prolonged hospital stays (OR 1.34, 95% CI 1.02—1.76; *P* = 0.003 for S1HR; OR 1.54, 95% CI 1.29—1.87; *P* = 0.01 for S2HR), with no significant difference between the high-risk groups (OR 1.03, 95% CI 0.79—1.34; *P* = 0.7).

**Conclusions:**

A high-risk for OSA, as identified by either the STOP-Bang or Step-2 assessment tools, is independently associated with an increased risk of postoperative complications and prolonged hospital length of stay. Both screening strategies demonstrated comparable predictive performance. These findings underscore the value of structured preoperative screening in identifying patients with a high likelihood of previously undiagnosed OSA and in guiding perioperative risk stratification and postoperative management.

**Supplementary Information:**

The online version contains supplementary material available at 10.1186/s12871-026-03900-1.

## Background

Obstructive Sleep Apnea (OSA) is characterized by recurrent episodes of partial or complete airway obstruction during sleep despite ongoing respiratory effort [1]. The disorder results from dynamic airway collapse during inspiration and is associated with repetitive hypoxia, hypercapnia, and sympathetic activation. Over time, these pathophysiological mechanisms contribute to the development of cardiovascular and metabolic comorbidities [1, 2].

In the perioperative setting, patients with OSA are at increased risk of postoperative complications due to the combined effects of underlying comorbidities and anesthesia-induced alterations in upper airway tone and ventilatory control. Sedatives, anesthetic agents, and opioids further exacerbate airway collapsibility and impair respiratory drive, thereby increasing the likelihood of respiratory and cardiovascular adverse events in the postoperative period [3–5].

Given the high yet frequently underestimated prevalence of OSA in the adult surgical population – of whom up to (70%) remain undiagnosed—routine preoperative screening is strongly recommended [6]. The most widely validated screening instrument is the STOP-Bang questionnaire, which stratifies patients according to their risk of moderate to severe OSA using the STOP-Bang Score (SBS). The score is derived from either eight clinical predictor variables and categorizes patients into three risk groups: low-risk (SBS 0—2) intermediate risk (SBS 3—4), and high-risk (SBS 5—8) [6]. While the STOP-Bang questionnaire demonstrates high sensitivity for identifying patients at risk, its specificity is moderate [6].

To enhance risk stratification among patients classified as intermediate risk by the STOP-Bang score and to identify additional high-risk individuals who may not be captured by the conventional STOP- Bang high-risk category, the so-called Step-2 algorithm was introduced. This algorithm builds upon the STOP-Bang score by incorporating additional stratification criteria to further refine risk assessment [6]. However, data on the perioperative implications and clinical utility of this extended screening approach remain limited.

The hypothesis of this study was that patients classified as high-risk for OSA by either screening strategy exhibit a higher incidence of postoperative complications and require more intensive perioperative management compared with low-risk patients. Furthermore, we hypothesized that the STOP-Bang questionnaire and the Step-2 scoring algorithm differ in their ability to identify patients at increased perioperative risk.

## Materials and methods

### Study design

The study was approved by the Ethics Committee of the University Medical Center Freiburg (AZ 164/19; June 6, 2019) without the consent of individual patients. Clinical trial registration was not applicable at the time. The study was conducted at a high-volume tertiary care university hospital (annual case volume ~ 100,000; case mix index ~ 1.3), performing approximately 35,000 surgical procedures requiring anesthesia per year.

**Fig. 1:**
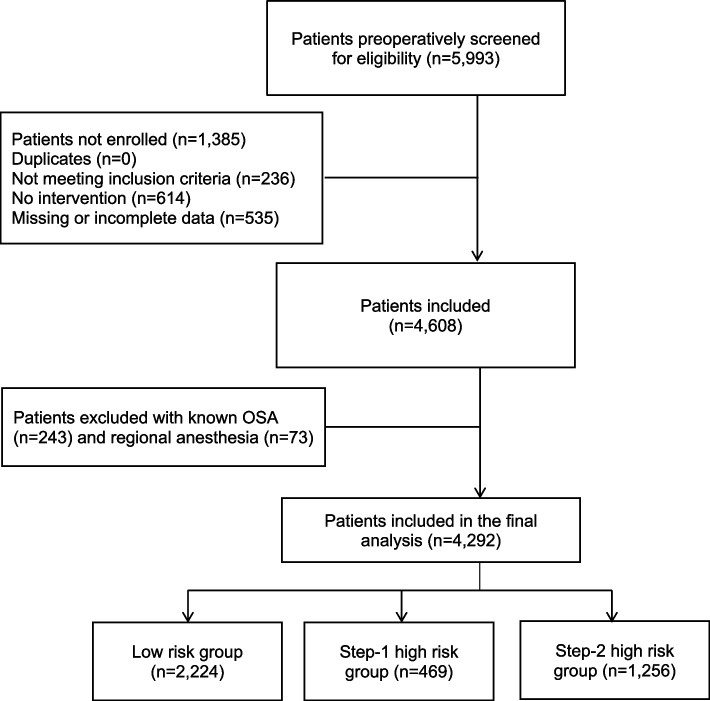
Consort diagram of inclusions, exclusions and participants available for analysis

Adult patients scheduled for elective non-cardiac surgery under sedation or general anesthesia between January 1, 2015, and May 1, 2016, were eligible for inclusion (see Fig. 1). Details regarding surgical procedures and anesthetic management are summarized in Table 2.

Preoperative assessment of OSA risk was performed routinely during the anesthesia consultation using the STOP-Bang score (SBS) and the Step-2 (S2) algorithm, with results documented in the anesthesia records.

The relevant data were extracted from electronic medical, anesthesiological and surgical records and collected in a database after pseudonymization:

- Preoperatively: age, sex, body mass index, classification according to the Physical Status Classification System of the American Society of Anesthesiologists, Revised Cardiac Risk Index, prevalence of comorbidities as assessed by the attending anesthesiologist.
- Intraoperatively: surgical department, duration of surgery, type of anesthesia.
- Postoperatively: transfer after surgery, length of hospital stays, pulmonary complications, cardiovascular complications.

Perioperative data were extracted from anesthesia records (MEDLINQ-EASY-Web, MEDLINQ software version UKFR 2013.1 KDS 3.0). Postoperative complications and hospital length of stay were obtained from administrative data (3 M™ QS-MED Suite) using relevant diagnostic and procedural codes based on the International Statistical Classification of Diseases and Related Health Problems (ICD-10) and the local procedure classification system.

Data were merged into a central dataset following a predefined data management scheme and categorized using electronic spreadsheets (Microsoft Excel for Mac 2011, Version 14.5.8). To minimize data transfer errors, multiple patient-specific identifiers were used to match records across datasets.

Only complete data sets were analyzed, patients with incomplete data were excluded. Patients with previously diagnosed OSA were excluded to avoid differences due to the diagnosis and resulting changes in postoperative care.

### Outcomes and measurements

The primary endpoint was the occurrence of major postoperative complications within the length of hospital stay. The composite outcome “*postoperative complications”* comprised postoperative pulmonary complications (PPC) and/or cardiovascular complications (CVC). Patients could experience more than one complication.

Postoperative pulmonary complications (PPC) were defined according to the European Perioperative Clinical Outcome (EPCO) criteria and included postoperative pneumonia, reintubation, postoperative aspiration, acute respiratory distress syndrome (ARDS) as defined by the Berlin definition, as well as new-onset respiratory failure requiring therapeutic non-invasive ventilation [7, 8].

Cardiovascular complications (CVC) included new-onset arrhythmia/atrial fibrillation, acute heart failure, acute myocardial ischemia/infarction, cardiac arrest and pulmonary embolism.

The secondary endpoint was the incidence of a prolonged hospital length of stay. Based on the mean length of hospital stay of 6.7 days in the study cohort, a hospital stay exceeding 7 days was defined as prolonged.

Patients were stratified according to their screening results into three groups: the low-risk (LR) group, comprising patients with a SBS of 0—2, the “S1HR” (Step-1 high-risk) group, including patients with an SBS of 5—8; and the “S2HR” (Step-2 high-risk) group, consisting of patients with an intermediate SBS of 3—4 in combination with at least one additional risk factor: body mass index (BMI) > 35 kg/m^2^, neck circumference > 40 cm, or male sex.

### Statistical analysis

A descriptive analysis comparing patients` intra- and postoperative characteristics was performed to identify differences between the groups. Categorical variables were presented as frequencies with proportions, and they were compared across groups using chi-square and Fisher exact test. Normal distribution was tested using the Shapiro-Wilks test. Continuous variables were presented as mean and standard deviation and compared with the students T-test or ANOVA (Analysis of Variance) with post hoc significance (Tukey`s test) across groups. To explore the association between the clinical outcomes and the different score groups, logistic regression models were built to calculate the odds ratio (OR) and the 95% confidence interval (CI). In the crude logistic regression analysis, the outcome as dependent variable and the assigned risk groups as independent variable were analyzed. To adjust for the impact of possible confounders, the multivariable model was adjusted for ASA PS, type and duration of surgery, type of anesthesia (regional anesthesia, sedation, general anesthesia, combined general and regional anesthesia), physical fitness (± 4 MET) and cardiovascular, respiratory, neurologic, and endocrine comorbidity, which were identified as differences between the groups. Age, arterial hypertension, BMI and gender were omitted since they are among the variables determining the STOP-Bang score. A Hosmer–Lemeshow test was performed to goodness-of-fit of the logistic regression models. To test for multicollinearity, the variance inflation factor was calculated, indicating a low likelihood of multicollinearity. All statistical analyses were performed using IBM SPSS Statistic for Windows (Version 23, Armonk, NY, USA, IBM Corp.). A *P* value of < 0.05 was considered significant. All tests were two-tailed.

To evaluate the association between the high-risk groups and other perioperative risk indicators (ASA-PS and RCRI), Spearman’s rank correlation coefficient (ρ) was calculated. The strength of correlation was interpreted as follows: 0–0.2, very weak; 0.2–0.4, weak; 0.4–0.6, moderate; and 0.6–0.8, strong.

## Results

### Study population

A total of 4,292 patients were included in the final analysis (Fig. 1), of whom 1,1774 (41.4%) were female and 2,518 (58.6%) were male.

Overall, 2,224 patients (51.8%) were classified as low-risk (LR, with a SBS 0—2). According to the one-step STOP-Bang score classification, 469 patients (10.9%) were categorized as high-risk (S1HR, SBS 5—8). Using the Step-2 algorithm, 1,256 patients (29.2%) were identified as high-risk (S2HR). Postoperative complications occurred in 335 patients (7.8%) within the overall cohort. Specifically, 107 patients (2.5%) developed postoperative pulmonary complications, whereas 245 patients (5.5%) experienced cardiovascular complications. The mean postoperative length of hospital stay for the entire cohort was 6.7 days (± 6.6).

Descriptive statistics stratified by risk groups are presented in Table 1.

**Table 1:** Demographic and patient characteristics

Variable	Overall	LR	S_1_ HR	S_2_ HR	*P*-Value		
	(*n* = 4,292)	(*n* = 2,224)	(*n* = 469)	(*n* = 1,256)	LR-S_1_ HR	LR-S_2_ HR	S_1_ HR- S_2_ HR
Age (Years) (SD)	53.9 (18.0)	49.1 (18.1)	63 (12)	60.8 (14.4)	< 0.001	< 0.001	< 0.001
Male sex, n (%)	2,518 (58.6)	1,017 (45.7)	377 (80.4)	1,124 (89.4)	< 0.001	< 0.001	< 0.001
BMI (kg/m^2^) (SD)	27.1 (5.9)	25.4 (4.4)	32.8 (7.7)	28.4 (6.3)	< 0.001	< 0.001	< 0.001
MET > 4, n (%)	3,830 (89.2)	2,110 (94.9)	365 (77.8)	1,110 (87.5)	< 0.001	< 0.001	< 0.001
ASA PS ≥ III n (%)	1,181 (27.5)	262 (11.8)	288 (61.4)	475 (37.8)	< 0.001	< 0.001	< 0.001
RCI, n (%) > = 2	155 (3.6)	16 (0.7)	46 (9.8)	70 (5.5)	< 0.001	< 0.001	< 0.001
Comorbidities, n (%)							
Arterial hypertension	1,695 (39.5)	288 (12.9)	415 (88.5)	701 (55.8)	< 0.001	< 0.001	< 0.001
Neurological disease	604 (14.1)	259 (11.6)	90 (14.9)	193 (15.5)	< 0.001	0.02	0.3
Coronary artery disease	286 (6.7)	43 (1.9)	76 (16.2)	133 (10.6)	< 0.001	< 0.001	0.02
Heart failure	190 (4.4)	32 (1.4)	40 (8.5)	89 (7.1)	< 0.001	< 0.001	0.23
Respiratory disease	656 (15.3)	238 (10.7)	126 (26.9)	235 (18.7)	< 0.001	< 0.001	< 0.001
Endocrine disease	897 (20.9)	355 (16)	145 (30.9)	285 (22.7)	< 0.001	< 0.001	0.001
RCI, n (%) > = 2	155 (3.6)	16 (0.7)	46 (9.6)	67 (5.3)	< 0.001	< 0.001	< 0.001

Descriptive statistics comparing the preoperative and demographic characteristics are shown as number of individuals (n), percentage (%) or mean ±. standard deviation

*Abbreviations*: *LR* Low-risk group, *S*_*1*_*HR* Step-1 high-risk group, *S*_*2*_*-HR* Step-2 high-risk group, *ASA PS* American Society of Anesthesiologists Physical Status, *BMI* Body mass index, *MET* Metabolic equivalent of task, *RCI* Revised cardiac risk index

### General demographic characteristics and comorbidities

Significant differences in demographic and clinical characteristics were observed between the low-risk group and both high-risk groups. Patients classified as high-risk were older, more frequently male, and more often assigned to higher ASA-PS categories. In addition, they exhibited a higher BMI and reduced functional capacity compared with low-risk patients (Table 1). Within the high-risk cohort, sex distribution differed between groups, with a higher proportion of male patients in the S2HR group. Patients classified as S1HR were older and had a higher BMI than those in the S2HR group. Furthermore, S1HR patients were more frequently categorized as ASA-PS III or higher and had a greater burden of comorbidities, except for neurological disease and heart failure. A smaller proportion of patients in the S1HR group achieved an exercise capacity exceeding 4 metabolic equivalents (METs).

### Intraoperative characteristics

Table 2 provides a detailed comparison of surgical procedures, anesthetic techniques, and duration of surgery across the different risk groups. The duration of surgery did not differ between groups. No significant differences were observed in the distribution of dermatological, ophthalmological, gynecological, or otorhinolaryngological procedures. In contrast, patients with a SBS of 0–2 more frequently underwent plastic, maxillofacial, and orthopedic surgery, whereas general and urological procedures were less common in this group compared with both high-risk groups. Between the high-risk cohorts, general surgical procedures were performed more frequently and urological procedures less frequently in the S1 HR group than in the S2 HR group. General anesthesia was the predominant anesthetic technique among high-risk patients, while the use of combined general and regional anesthesia was less frequent. Importantly, neither avoidance of general anesthesia nor preferential use of regional was observed in the high-risk groups compared with the low-risk group.

**Table 2:** Intraoperative characteristics

Variable	Overall	LR	S_1_ HR	S_2_ HR	P-Value		
	(*n* = 4,292)	(*n* = 2,224)	(*n* = 469)	(*n* = 1,256)	LR-S_1_ HR	LR-S_2_ HR	S_1_ HR- S_2_ HR
Type of surgery, n (%)							
Orthopedic	1,924 (44.8)	1,159 (52.1)	149 (31.8)	475 (37.8)	< 0.001	< 0.001	0.05
Plastic	325 (7.6)	239 (10.7)	16 (3.5)	47 (3.7)	< 0.001	< 0.001	0.8
Dermatologic	194 (4.5)	102 (4.6)	26 (5.5)	57 (4.5)	0.6	0.9	0.6
Ophthalmic	733 (17.1)	339 (15.2)	85 (18.1)	207 (16.5)	0.25	0.1	0.9
Maxillofacial	189 (4.4)	128 (5.8)	10 (2.1)	41 (3.3)	< 0.001	0.01	0.1
Gynecologic	14 (0.3)	11 (0.5)	1 (0.2)	1 (0.1)	0.5	0.7	1
ENT	51 (1.2)	13 (0.6)	9 (1.9)	18 (1.4)	0.1	0.6	0.6
Urologic	603 (14.1)	175 (7.7)	98 (20.9)	305 (23.9)	< 0.001	< 0.001	0.04
General	207 (4.8)	47 (2.1)	62 (13.2)	87 (6.8)	< 0.001	< 0.001	< 0.001
other	52 (1.1)	11 (0.5)	13 (2.1)	18 (1.2)	< 0.001	0.8	0.8
Type of anesthesia, n (%)							
Sedation	212 (4.6)	97 (4.3)	26 (3.9)	70 (5.3)	0.74	0.16	0.18
GA	3,480 (78.2)	1,770 (78.2)	392 (81.7)	1047 (82.1)	0.05	0.01	0.2
Combined RA/GA	610 (14)	357 (15.8)	58 (12.1)	142 (11.1)	0.01	< 0.001	0.4
Duration of surgery (min)							
	76.2 (72.4)	76.7 (75.1)	77.0 (64)	76.0 (75.3)	0.5	0.4	0.6

Descriptive statistics comparing the intraoperative characteristics are shown as number of individuals (n), percentage (%) or mean ±. standard deviation

*Abbreviations*: *LR* Low-risk group, *S*_*1*_* HR* Step-1 high-risk group, *S*_*2*_* HR* Step-2 high-risk group, *ENT* Ear-nose-throat, *GA* General anesthesia, *RA* Regional anesthesia

### OSA risk assessment and postoperative complications

The proportion of patients requiring admission to an intensive care or high-density unit was highest in the S1HR group (18.3%), followed by the S2HR group (9.9%) and the low-risk group (4.6%).

Patients classified as low-risk had the shortest postoperative hospital stay, with a mean duration of 5.8 ± 5.7 days (Table 3). Length of stay was comparable between the two high-risk groups (S1HR: 8 ± 7.3 days; S2HR: 7.4 ± 7.5 days). The proportion of patients with a prolonged hospital stay (> 7 days) was significantly higher in both high-risk groups, with highest percentage observed in the S1HR group (LR: 529 patients [23.8%]; S1HR: 188 patients [40.1%]; S2HR: 459 patients [35%]; *P* < 0.001) (Table 3).

**Table 3:** Postoperative parameters and complications

Variable	Overall	LR	S_1_HR	S_2_HR	*p*-value		
	(*n* = 4,292)	(*n* = 2,224)	(*n* = 469)	(*n* = 1,256)	LR-S_1_HR	LR-S_2_HR	S_1_HR- S_2_HR
Postoperative admission destination							
PACU	3,382 (79.1)	1,891 (85.0)	318 (67.8)	898 (71.6)	< 0.001	< 0.001	0.01
Ward	534 (12.5)	218 (9.8)	63 (11.8)	227 (18)	0.02	0.01	0.01
ICU/HDU	352 (8.2)	103 (4.6)	86 (18.3)	124 (9.9)	< 0.001	< 0.001	< 0.001
other	24 (0.6)	12 (0.5)	2 (0.3)	7 (0.6)	0.3	0.3	0.9
hospital stay (days)	6.7 (6.6)	5.8 (5.7)	8 (7.3)	7.4 (7.5)	< 0.001	< 0.001	0.4
hospital stay > 7 days	1,312 (30.6)	529 (23.8)	188 (40.1)	459 (35)	< 0.001	< 0.001	0.02
Patients with Complications	335 (7.7)	74 (3.3)	77 (16.4)	154 (12.3)	< 0.001	< 0.001	0.01
PPC	107 (2.5)	33 (1.5)	26 (5.4)	38 (3)	< 0.001	0.004	0.02
CVC	245 (5.5)	47 (2.1)	61 (13)	124 (9.9)	< 0.001	< 0.001	0.02
Myocardial infarction	7 (0.2)	1	1 (0.2)	4 (0.3)			
Angina pectoris	10 (0.2)	4 (0.2)	2 (0.4)	3 (0.2)			
Heart failure	20 (0.4)	4 (0.2)	7 (1.4)	6 (0.4)			
Pulmonary embolism	12 (0.3)	2 (0.1)	2 (0.4)	6 (0.5)			
Arrhythmia	46 (1.0)	13 (0.6)	10 (2.1)	15 (1.2)			
Cardiac arrest	4 (0.1)	2 (0.1)	0	2 (0.2)			
Atrial fibrillation	173 (4)	22 (1)	41 (8.5)	90 (7.1)			

Postoperative complications occurred in 74 patients (3.4%) in the low-risk group, compared with 77 patients (16.4%) in the S1HR group and 154 patients (12.1%) in the S2HR group, representing a significantly higher complication rate in both high-risk groups (*P* < 0.001 for each comparison) (Table 3).

**Fig. 2:**
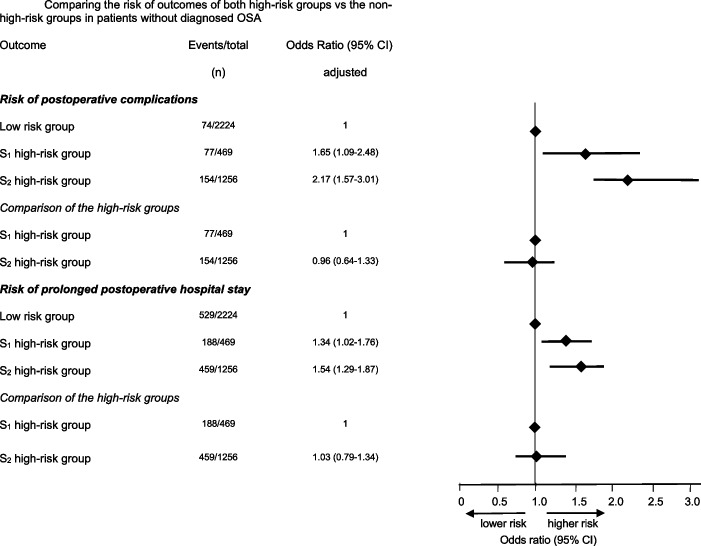
Comparing the risk of outcomes of both high-risk vs. the non-high-risk groups in patients without diagnosed OSA

In the univariate analysis, both high-risk groups demonstrated a significantly increased rate and risk of postoperative complications compared with the low-risk group (S1HR vs. low-risk: OR 5.71, 95% CI 4.08—7.99; S2HR vs. low-risk: OR 4.06 (95% CI 3.05—5.40) (Fig. 2). This association remained statistically significant after adjustment for potential confounders: S1HR vs. low-risk: OR: 1.65 (95% CI 1.09—2.48); S2HR vs. low-risk: OR 2.17 (95% CI 1.57—3.01)).

When the two high-risk groups were combined into a single high-risk category, these patients exhibited a significantly higher likelihood of experiencing postoperative complications (unadjusted OR: 4.49 (95% CI 3.43—5.88); adjusted OR: 2.11 (95% CI 1.55—2.86) as well as a prolonged hospital stay (unadjusted OR: 1.92 (95% CI 1.67—2.21); adjusted OR: 1.56 (95% CI 1.31—1.86) compared to the low-risk group (Fig. 2).

Direct comparison between the two high-risk groups showed a lower probability of postoperative complications in the S2HR group only in the unadjusted analysis (OR: 0.71 (95% CI 0.53—0.96). After adjusting for confounders, no significant difference was observed between S1HR and S2HR (Fig. 2).

The correlation between the two STOP-BANG high risk scores (S_1_HR, S_2_HR) and the ASA-PS and RCRI were each examined using the Spearman's *ρ* rank correlation coefficient; statistically significant (*P* < 0.001) though weak correlations were observed between S_2_-HR and the ASA-PS (*ρ* = 0.371) and RCRI (*ρ* = 0.264), and correlation was found to be moderate between S_1_HR and the ASA-PS (*ρ* = 0.45) (*p* < 0.001) and weak correlation between S_1_HR and RCRI (*ρ* = 0.34) (*p* < 0.001). The correlation coefficient between the joint-HR-group with ASA (*ρ* = 0.44) (*p* < 0.001) was moderate, with RCRI (0.28) the correlation was rather weak (*p* < 0.001).

## Discussion

### Outcomes and relevance

Our study demonstrates that patients identified as high-risk for moderate to severe obstructive sleep apnea (OSA) by both the STOP-Bang and Step-2 algorithms exhibit an increased risk of postoperative complications compared with those classified as low-risk.

Previous investigations assessing the relationship between the likelihood of OSA, as determined by the STOP-Bang questionnaire, and postoperative complications have produced inconsistent results [9–11]. Differences in outcome definitions, study populations, and methods of OSA classifications—ranging from questionnaire-based risk assessment to polysomnographic confirmation in already diagnosed patients—have complicated direct comparisons [12, 13]. Evidence regarding the correlation between Step-2 risk assessment and postoperative complications is scarce. Notably, the Step-2 algorithm demonstrates lower sensitivity, but higher specificity compared with the conventional STOP-Bang score, making it particularly useful for identifying additional patients at high risk of OSA [14]. A novel aspect of our study is the combined assessment of patients using both scoring systems, including those who may have been overlooked by Step-1 of the STOP-Bang score, thereby providing a more comprehensive evaluation of perioperative risk. Of note, while the high-risk group defined by Step-1 had a higher BMI, less patients in this group were male compared to the Step-2 high risk group, signifying the predictive value of both items.

We attempted to minimize treatment-related bias by excluding patients who had previously been diagnosed with OSA and were receiving specific therapies, such as continuous positive airway pressure (CPAP) [15, 16]. Employing the questionnaire alone provides a simple and standardized approach applicable to all patients. However, relying solely on screening questionnaires—originally not designed to predict postoperative complications and characterized by moderate specificity—may result in substantial proportion of false negatives, a limitation inherent to many screening tools.

Although OSA is primarily a respiratory disorder, respiratory complications occurred less frequently than cardiovascular complications in both our and previous studies [12, 17, 18]. Some authors attribute the higher incidence of cardiovascular complications, particularly those detected on the ward, to preceding episodes of unrecognized hypoxia [9]. This hypothesis could not be confirmed with our data and remains an area for further investigation.

### Comorbidities

Comorbidities are more common in patients with OSA and substantially contribute to increased perioperative risk [19]. In our cohort, significant differences in comorbidity profiles were observed between the two high-risk groups, which may account for the lower incidence of postoperative complications and shorter hospital stays in the Step-2 high-risk group in the univariate analysis. However, after adjustment for relevant comorbidities, the differences were no longer statistically significant, indicating that comorbidity burden largely explains the initially observed disparities between the high-risk groups. Given the pronounced differences between patients classified as low- and high-risk OSA, our findings suggest that OSA-related risk is at least partially independent of comorbidities. This interpretation is indirectly supported by previous studies demonstrating that adequate treatment of OSA and recognition of the diagnosis for perioperative risk stratification are associated with reduced postoperative complications [12, 20–22]. Nevertheless, the optimal intensity and duration of enhanced postoperative monitoring and targeted interventions in patients with suspected or untreated OSA remain to be defined. At our institution, as in many others, patients with suspected or untreated OSA are routinely managed postoperatively in a high-dependency or intensive care setting and, in the absence of complications, subsequently transferred to a step-down unit on the following postoperative day.

Postoperative exacerbation of OSA typically peaks around the third night and may persist until approximately the seventh night. This temporal pattern raises the question of whether prolonged and intensified postoperative surveillance in selected patients could reduce the incidence of complications. However, such approach is often impractical due to limited human and financial resources within healthcare system [13]. In this context, a stepwise and risk-adapted management strategy appears reasonable. Following the identification of patients with a high likelihood of OSA in the absence of prior polysomnographic evaluation, continuous postoperative respiratory monitoring may represent a practical initial measure. Modalities such as pulse oximetry or end-tidal capnography could facilitate early detection of critical respiratory events and prompt timely escalation of care. Preventive interventions, including supplemental oxygen, temporary CPAP, or other anti-obstructive therapies in the postoperative period, have demonstrated promising results and may constitute effective strategies in selected patients. Nevertheless, limited patient tolerance and adherence often restrict their universal applicability [23, 24]. Despite the associated logistical and economic burden, preoperative polysomnographic testing may be justified in purely elective cases to confirm the diagnosis and to identify specific OSA phenotypes, thereby enabling more individualized perioperative management strategies [25].

Another potential strategy involves tailoring the selection and modification of anesthetic and surgical techniques. However, the routine use of regional anesthesia to avoid the effects of general anesthesia is not always feasible and does not fully mitigate the perioperative risks associated with OSA [26–28]. In particular, the requirement for intensified and/or prolonged postoperative opioid analgesia or the use of centrally acting sedative agents represents a well-established risk factor for postoperative respiratory and cardiovascular complications and should therefore be explicitly addressed in institutional perioperative management protocols [7, 29]. To date, no anesthetic technique has been shown to be unequivocally superior in patients with OSA. Consequently, various anesthetic and surgical approaches may be appropriate, if they are accompanied by adequate postoperative monitoring and risk-adapted care pathways [8, 13, 15, 28]. A key future challenge will be the identification of individual risk factors and perioperative parameters that allow for more precise stratification and personalization of postoperative monitoring and treatment strategies.

### Limitations of study

A diagnosis of obstructive sleep apnea (OSA) cannot be established solely based on screening instruments such as the STOP-Bang questionnaire. A positive screening result requires confirmation by polysomnography or another objective diagnostic modality. Accordingly, the inherent limitations of screening tools—including their limited specificity and the potential overestimation of patients at risk—must be acknowledged. To specifically assess the predictive value of questionnaire-based risk stratification, we excluded patients with a previously established diagnosis of OSA and/or ongoing OSA-specific treatment. This may affect the cross-sectional construct validity of the study, as most excluded patients were classified as S1 rather than S2 high-risk. Furthermore, patients with a prior diagnosis of obstructive sleep apnea may have undergone intensified perioperative monitoring and management, potentially influenced postoperative outcomes and introduced bias within the high-risk groups. The use of screening questionnaires alone offers a pragmatic and resource-efficient approach, as it does not necessitate additional diagnostic testing associated with substantial logistical, financial, and organizational burdens.

Age and body mass index were not included as separate covariates in the multivariable model, as they are integral components of the risk scores under investigation. While this approach avoids potential overadjustment, it limits the ability to disentangle the independent effects of these individual variables from the overall score performance.

Nevertheless, we cannot exclude the possibility that patients who developed clinical symptoms in the postoperative period received intensified monitoring or additional therapeutic interventions, which may have influenced outcomes. The screening questionnaires used in this study are characterized by limited predictive accuracy. Assessing the discriminative performance (see supplementary data), their contribution is rather based on the negative predictive value. As their primary purpose is to stratify the risk of obstructive sleep apnea rather than to predict postoperative complications, an exact prediction of postoperative adverse events cannot be expected. This was confirmed measuring the correlation coefficients with ASA PS and RCRI as further perioperative risk tools. The relatively weak correlation may be explained by the different purpose: STOP-BANG was created as a screening tool for OSA 10; ASA-PS to assess perioperative health, Revised Cardiac Risk Index to assess the risk of perioperative major adverse cardiac events. The aim of this study was therefore not to establish a causal prediction model for postoperative complications, but to demonstrate that patients with high screening scores are at an increased risk and that the Step-2 algorithm may improve the identification of patients with a high likelihood of OSA. Based on the screening results, our data suggest a prevalence of mild to severe OSA of approximately 40% within the cohort. This estimate appears high and is likely influenced by the limited specificity of the screening tools, leading to an overestimation of true disease prevalence. Information on the actual prevalence of OSA confirmed by objective diagnostic testing (e.g., polysomnography) would have been highly informative; however, such data were not available in this cohort.

Postoperative complications were assessed over the entire hospital stay. Consequently, events occurring later in the postoperative course may be less directly attributable to the immediate effects of surgery and anesthesia. To facilitate clarity of data presentation and interpretation, we focused on comparing the two high-risk groups with an established low-risk group and their associations with postoperative complications and length of hospital stay. Although the study reflects a real-world clinical population, the single-center design and distribution of surgical specialties may limit generalizability. Future multicenter studies with a more balanced case mix are warranted to confirm these findings.

## Conclusion

Our findings support the utility of both the STOP-Bang score and the Step-2 algorithm in identifying patients at increased risk for postoperative complications. Incorporation of these screening tools into routine perioperative assessment may facilitate risk stratification and inform individualized perioperative management, including the adaptation of anesthetic and surgical strategies according to the patient’s risk profile. Moreover, these scores may assist in determining the appropriate level of postoperative monitoring and in planning the anticipated length of hospital stay. Further prospective studies are warranted to evaluate whether risk-adapted perioperative care strategies based on OSA screening translate into improved clinical outcomes.

## Supplementary Information

Supplementary Material 1.

## Data Availability

The datasets used and analyzed during the current study are available from the corresponding author on request.
