# Supplementary material for: Obstructive sleep apnea (OSA) screening and risk of postoperative complications in adult surgical patients: differences between the STOP-Bang and Step-2 scoring strategy
Source: BMC Anesthesiol. 2026 May 13;26:301. doi: 10.1186/s12871-026-03900-1 (PMC13173807; doi:10.1186/s12871-026-03900-1)
Supplement: Supplementary file 1 — Supplementary Material 1. [file 12871_2026_3900_MOESM1_ESM.docx]

**Supplementary Data**

**Tables presenting the full cohort including those preclassified “intermediate risk”**

| **Table S1** |  |  |  |  |  |  |
| --- | --- | --- | --- | --- | --- | --- |
| **Variable** | | **Overall** | **LR** | **IR** | **S_1_ HR** | **S_2_ HR** |
|  |  | (n=4,292) | (n=2,224) | (n=342) | (n=469) | (n=1,256) |
| Age (Years) (SD) | | 53.9 (18.0) | 49.1 (18.1) | 68.1 (10.8) | 63 (12) | 60.8 (14.4) |
| Male sex, n (%) | | 2,518 (58.6) | 1,017 (45.7) | 0 | 377 (80.4) | 1,124 (89.4) |
| BMI (kg/m^2^) (SD) | | 27.1 (5.9) | 25.4 (4.4) | 27.1 (4.4) | 32.8 (7.7) | 28.4 (6.3) |
| MET>4, n (%) | | 3,830 (89.2) | 2,110 (94.9) | 255 (74.6) | 365 (77.8) | 1,110 (87.5) |
| ASA PS ≥III n (%) | | 1,181 (27.5) | 262 (11.8) | 156 (45.5) | 288 (61.4) | 475 (37.8) |
| **Comorbidities, n (%)** | |  |  |  |  |  |
| Arterial hypertension | | 1,695 (39.5) | 288 (12.9) | 291 (85.1) | 415 (88.5) | 701 (55.8) |
| Neurological disease | | 604 (14.1) | 259 (11.6) | 62 (20.3) | 90 (14.9) | 193 (15.5) |
| Coronary artery disease | | 286 (6.7) | 43 (1.9) | 34 (9.9) | 76 (16.2) | 133 (10.6) |
| Heart failure | | 190 (4.4) | 32 (1.4) | 29 (8.4) | 40 (8.5) | 89 (7.1) |
| Respiratory disease | | 656 (15.3) | 238 (10.7) | 57 (16.6) | 126 (26.9) | 235 (18.7) |
| Endocrine disease | | 897 (20.9) | 355 (16) | 112 (32.7) | 145 (30.9) | 285 (22.7) |
| RCRI, n (%) >=2 | | 155 (3.6) | 16 (0.7) | 26 (7.6) | 46 (9.6) | 67 (5.3) |

Table S1. Descriptive statistics comparing the preoperative and demographic characteristics are shown as number of individuals (n), percentage (%) or mean ± standard deviation. Abbreviations: LR, Low-risk group; IR, intermediate risk according to Step-2; S_1_HR; Step-1 high-risk group; S_2_-HR: Step-2 high-risk group, ASA PS: American Society of Anesthesiologists Physical Status; BMI: body mass index; MET: metabolic equivalent of task; RCRI: revised cardiac risk index.

| **Table S2** |  |  |  |  |  |  |
| --- | --- | --- | --- | --- | --- | --- |
| **Variable** | | **Overall** | **LR** | **IR** | **S_1_ HR** | **S_2_ HR** |
|  |  | (n=4,292) | (n=2,224) | (n=342) | (n=469) | (n=1,256) |
| Type of surgery, n (%) | |  |  |  |  |  |
| Orthopedic | | 1,924 (44.8) | 1,159 (52.1) | 141 (41.2) | 149 (31.8) | 475 (37.8) |
| Plastic | | 325 (7.6) | 239 (10.7) | 23 (6.7) | 16 (3.5) | 47 (3.7) |
| Dermatologic | | 194 (4.5) | 102 (4.6) | 9 (2.6) | 26 (5.5) | 57 (4.5) |
| Ophtalmic | | 733 (17.1) | 339 (15.2) | 102 (29.8) | 85 (18.1) | 207 (16.5) |
| Maxillofacial | | 189 (4.4) | 128 (5.8) | 10 (2.9) | 10 (2.1) | 41 (3.3) |
| Gynecologic | | 14 (0.3) | 11 (0.5) | 1 (0.3) | 1 (0.2) | 1 (0.1) |
| ENT | | 51 (1.2) | 13 (0.6) | 11 (3.2) | 9 (1.9) | 18 (1.4) |
| Urologic | | 603 (14.1) | 175 (7.7) | 29 (8.5) | 98 (20.9) | 305 (23.9) |
| General | | 207 (4.8) | 47 (2.1) | 13 (3.8) | 62 (13.2) | 87 (6.8) |
| other | | 52 (1.1) | 11 (0.5) | 10 (3.3) | 13 (2.1) | 18 (1.2) |
| Type of anesthesia | |  |  |  |  |  |
| Sedation | | 212 (4.6) | 97 (4.3) | 19 (5.7) | 26 (3.9) | 70 (5.3) |
| General | | 3,480 (78.2) | 1,770 (78.2) | 271 (79.2) | 392 (81.7) | 1047 (82.1) |
| Combined RA/GA | | 610 (14) | 357 (15.8) | 53 (15.5) | 58 (12.1) | 142 (11.1) |
| Duration surgery (min) | | 76.2 (72.4) | 76.7 (75.1) | 75.9 (69) | 77.0 (64) | 76.0 (75.3) |

Table S2. Descriptive statistics comparing the intraoperative characteristics are shown as number of individuals (n), percentage (%) or mean ±. standard deviation. Abbreviations: LR, Low-risk group; IR, intermediate risk according to Step2; S_1_ HR; Step-1 high-risk group; S_2_ HR: Step-2 high-risk group; ENT: ear-nose-throat; GA: general anesthesia; RA: regional anesthesia.

**Table S3**

| **Variable** | **Overall** | | **LR** | | **IR** | | **S_1_ HR** | | **S_2_ HR** | |
| --- | --- | --- | --- | --- | --- | --- | --- | --- | --- | --- |
|  | (n=4,292) | | (n=2,224) | | (n=342) | | (n=469) | | (n=1,256) | |
| PACU | 3,382 (79.1) | 1,891 (85.0) | | 272 (79.5) | | 318 (67.8) | | 898 (71.6) | |  |
| Ward | 534 (12.5) | 218 (9.8) | | 28 (8.2) | | 63 (11.8) | | 227 (18) | |  |

| ICU/HDU | 352 (8.2) | 103 (4.6) | 39 (11.4) | 86 (18.3) | 124 (9.9) |
| --- | --- | --- | --- | --- | --- |
| other | 24 (0.6) | 12 (0.5) | 3 (1) | 2 (0.3) | 7 (0.6) |
| hospital stay (days) | 6.7 (6.6) | 5.8 (5.7) |  | 8 (7.3) | 7.4 (7.5) |
| hospital stay >7days | 1,312 (30.6) | 529 (23.8) | 136 (39.8) | 188 (40.1) | 459 (35) |

| Patients with complications | 335 (7.7) | 74 (3.3) | 30 (8.7) | 77 (16.4) | 154 (12.3) |
| --- | --- | --- | --- | --- | --- |
| PPC | 107 (2.5) | 33 (1.5) | 10 (2.9) | 26 (5.4) | 38 (3) |
| Pneumonia | 14 (0.3) | 3 (0.1) | 1 (0.3) | 4 (0.9) | 6 (0.5) |

| Reintubation | 11 (0.3) | 0 | 1 (0.3) | 4 (0.9) | 6 (0.5) |
| --- | --- | --- | --- | --- | --- |
| Pulmonary aspiration | 8 (0.2) | 2 (0.1) | 1 (0.3) | 2 (0.5) | 3 (0.2) |
| ARDS | 11 (0.3) | 3 (0.1) | 1 (0.3) | 4 (0.9) | 3 (0.2) |
| Respiratory failure | 92 (2.1) | 26 (1.2) | 12 (3.5) | 22 (4.7) | 32 (2.5) |
| CVC | 245 (5.7) | 47 (2.1) | 13 (3.8) | 61 (13) | 124 (9.9) |
| Myocardial infarction | 9 (0.2) | 2 (0.1) | 1 (0.3) | 1 (0.2) | 5 (0.4) |
| Angina pectoris | 12 (0.3) | 5 (0.2) | 0 | 2 (0.4) | 5 (0.5) |
| Heart failure | 22 (0.4) | 4 (0.2) | 3 (0.9) | 7 (1.4) | 8 (0.6) |
| Pulmonary embolism | 16 (0.4) | 3 (0.1) | 2 (0.6) | 4 (0.9) | 7 (0.6) |
| Cardiac arrest | 4 (0.1) | 2 (0.1) | 0 | 0 | 2 (0.2) |
| Atrial fibrillation | 201 (4.6) | 36 (1.6) | 7(2) | 54 (11.5) | 104 (8.3) |

| RCRI, n (%) >=2 | 155 (3.6) | 16 (0.7) | 26 (7.6) | 46 (9.6) | 67 (5.3) |
| --- | --- | --- | --- | --- | --- |

Table S3: Descriptive statistics comparing the postoperative parameters and complications. Data are shown as number of individuals (n) or percentage (%). Abbreviations: LR: low-risk group; IR, intermediate risk according to Step2; S_1_HR: Step-1 high-risk group; S_2_-HR: Step-2 high-risk group; PACU: post-anesthesia care unit; ICU/HDU: intensive/high-density care unit; PPC: postoperative pulmonary complications; CVC: cardiovascular complications.

**Table S4: Comparing the risk of outcomes of both high-risk groups vs the non-high-risk groups in patients without diagnosed OSA**

| \| Outcome \| Odds Ratio (95% CI) \| Odds Ratio (95% CI) \| r^2^ (Nagelkerkes) \| Hosmer-Lemeshow Goodness-of-Fit \| X^2^ \| df \| \| --- \| --- \| --- \| --- \| --- \| --- \| --- \| \| unadjusted \| adjusted \|  \|  \|  \|  \| \| ***Risk of postoperative complications*** \| \| \| \| \| \| \| \| Low risk \| 1 \| 1 \|  \|  \|  \|  \| \| S_1_ high-risk \| 5.71 (4.1-7.9) \| 1.7 (1.1-2.5) \| 0.312 \| 0.438 \| 7.9 \| 8 \| \| S_2_ high-risk \| 4.1 (3.1-5.4) \| 2.2 (1.6-3.0) \| 0.262 \| 0.694 \| 5.6 \| 7 \| \| *Comparison of the high-risk groups* \| \| \| \| \| \| \| \| S_1_ high-risk \| 1 \| 1 \|  \|  \|  \|  \| \| S_2_ high-risk \| 0.7 (0.5-0.96) \| 0.96 (0.6-1.3) \| 0.223 \| 0.157 \| 11.86 \| 8 \| \| ***Risk of prolonged postoperative hospital stay*** \| \| \| \| \| \| \| \| Low risk \| 1 \| 1 \|  \|  \|  \|  \| \| S_1_ high-risk \| 2.1 (1.7-2.6) \| 1.3 (1.0-1.8) \| 0.305 \| 0.144 \| 12.1 \| 8 \| \| S_2_ high-risk \| 1.8 (1.6-2.1) \| 1.5 (1.3-1.9) \| 0.332 \| 0.396 \| 16.3 \| 8 \| \| *Comparison of the high-risk groups* \| \| \| \| \| \| \| \| S_1_ high-risk \| 1 \| 1 \|  \|  \|  \|  \| \| S_2_ high-risk \| 0.9 (0.7-1.1) \| 1.0 (0.8-1.3) \| 0.362 \| 0.157 \| 11.9 \| 0.16 \| | |
| --- | --- | --- | --- | --- | --- | --- | --- | --- | --- | --- | --- | --- | --- | --- | --- | --- | --- | --- | --- | --- | --- | --- | --- | --- | --- | --- | --- | --- | --- | --- | --- | --- | --- | --- | --- | --- | --- | --- | --- | --- | --- | --- | --- | --- | --- | --- | --- | --- | --- | --- | --- | --- | --- | --- | --- | --- | --- | --- | --- | --- | --- | --- | --- | --- | --- | --- | --- | --- | --- | --- | --- | --- | --- | --- | --- | --- | --- | --- | --- | --- | --- | --- | --- | --- | --- | --- | --- | --- | --- | --- | --- | --- | --- | --- | --- | --- | --- | --- | --- | --- | --- | --- | --- | --- | --- | --- | --- | --- | --- | --- | --- | --- |
| Tables S4. Comparing the risk of postoperative complications and prolonged hospital stay between the different groups. Abbreviations: OR: odds ratio; CI: confidence interval; df: degree of freedom; LR: low risk; S_1_HR: Step-1 high-risk group; S_2_-HR: Step-2 high-risk group. |  |
|  |  |

| **Table S5: Comparing the risk of outcomes of both high-risk groups vs the non-high-risk groups in patients without**  **diagnosed OSA** |  |  |  |  |  |  |
| --- | --- | --- | --- | --- | --- | --- |
| \| Outcome \| Odds Ratio  (95% CI) \| Odds Ratio  (95% CI) \| r^2^ (Nagelkerkes) \| Hosmer-Lemeshow \| \| X^2^ \| \| df \| \| \| --- \| --- \| --- \| --- \| --- \| --- \| --- \| --- \| --- \| --- \| \| unadjusted \| adjusted \|  \|  \| \|  \| \|  \| \| \| ***Risk of postoperative complications*** \| \| \| \| \| \| \| \| \| \| \| *Joint high-risk groups vs. non-high-risk groups* \| \| \| \| \| \| \| \| \| \| \| Low-risk \| 1.0 (1.0-1.0) \| 1.0 (1.0-1.0) \|  \| \|  \| \|  \| \|  \| \| High-risk \| 4.5 (3.4-5.9) \| 2.1 (1.6-2.9) \| 0.289 \| \| 0.428 \| \| 8.06 \| \| 8 \| \| ***Risk of prolonged postoperative hospital stay*** \| \| \| \| \| \| \| \| \| \| \| *Joint high-risk groups vs. non-high-risk groups* \| \| \| \| \| \| \| \| \| \| \| Low-risk \| 1 \| 1 \|  \| \|  \| \|  \| \|  \| \| High-risk \| 1.9 (1.7-2.2) \| 1.6 (1.3-1.9) \| 0.321 \| \| 0.09 \| \| 18.23 \| \| 8 \| |  |  |  |  |  |  |
| Tables S5. Comparing the risk of postoperative complications and prolonged hospital stay between the different groups. Abbreviations: OR: odds ratio; CI: confidence interval; df: degree of freedom. |  |  |  |  |  |  |

**Table S6**

|  | AUROC | 95% CI | p-value | Sensitivity (%) | Specficity (%) | PPV  (%) | NPV  (%) |
| --- | --- | --- | --- | --- | --- | --- | --- |
| **Complications** | | | | | | | |
| S_1_HR | 0.678 | 0.63-0.73 | 0.025 | 51  (42.7-59.2) | 84.5  (83.12-85.9) | 21.6  (18.7-24.8) | 82  (80.5-83.4) |
| S_2_HR | 0.668 | 0.63-0.70 | 0.02 | 67.5  (61.1-73.6) | 66.1  (64.5-67.8) | 14.3  (13.1-15.5) | 96.1  (95.3-96.7) |
| jHR | 0.674 | 0.644-0.703 | 0.015 | 75.7  (70.5-80.4) | 59  (57.4-60.6) | 13.35  (12.5-14.2) | 96.7  (95.9-97.3) |
| **Prolonged hospital stay** | | | |  |  |  |  |
| S_1_HR | 0.56 | 0.536-0.59 | 0.013 | 26.2  (23.0-29.6) | 85.8  (84.2-87.3) | 44.1  (40.1-48.2) | 73.1  (72.1-74) |
| S_2_HR | 0.572 | 0.55-0.59 | 0.011 | 46.5  (43.3-49.7) | 68.0  (66.1-69.9) | 38.4  (36.3-40.5) | 74.8  (73.6-75.9) |
| jHR | 0.581 | 0.56-0.60 | 0.01 | 55  (52.1-57.9) | 69.6  (67.7-71.4) | 43.7  (41.7-45.6) | 78.3  (77.1-79.5) |

Table S6. Both high-risk scores and the joint high-risk group showed rather moderate discrimination for postoperative complications. The area under the receiver operating curve (AUROC) including 95% confidence interval, sensitivity and specificity, positive and negative predictive value are reported. Abbreviations: AUROC: area under receiving operator curve; S_1_HR: Step-1 high-risk group; S_2_-HR: Step-2 high-risk group; jHR: joint high-risk group; CI: confidence interval.

**Concurrent construct validity**

The correlation between the two STOP-BANG high risk scores (S_1_HR, S_2_HR) and the ASA-PS and RCRI were each examined using the Spearman's *ρ* rank correlation coefficient; statistically significant (*P*<0.001) though weak correlations were observed between S_2_-HR and the ASA-PS (*ρ*=0.371) and RCRI (*ρ*=0.264), and correlation was found to be moderate between S_1_HR and the ASA-PS (*ρ*=0.45) (p<0.001) and weak correlation between S_1_HR and RCRI (*ρ*=0.34) (p<0.001). The correlation coefficient between the joint-HR-group with ASA (*ρ*=0.44) (p<0.001) was moderate, with RCRI (0.28) the correlation was rather weak(p<0.001).
